# General Expression for Vibronic Coupling in Proton-Coupled Energy Transfer

**DOI:** 10.1021/acs.jctc.6c00443

**Published:** 2026-05-24

**Authors:** Kai Cui, Sharon Hammes-Schiffer

**Affiliations:** Department of Chemistry, 6740 Princeton University, Princeton, New Jersey 08544, United States

## Abstract

Proton-coupled energy transfer (PCEnT) is a recently discovered process in which electronic excitation energy transfer is coupled to proton transfer. In nonadiabatic PCEnT theory, the reaction is described in terms of nonadiabatic transitions between pairs of electron–proton vibronic states, and the PCEnT rate constant associated with each pair depends on the square of the coupling between the reactant and product vibronic states. Herein, we derive an analytical expression for the diabatic vibronic coupling in PCEnT processes. The resulting total vibronic coupling encompasses both direct and indirect coupling between the reactant and product vibronic states. The direct coupling includes both Coulomb and exchange interactions between the reactant and product vibronic states, whereas the indirect coupling describes the interaction mediated by virtual intermediate vibronic states. The relative magnitudes of these interactions determine whether the Förster or Dexter mechanism for energy transfer is dominant, or whether a more general mechanism prevails. Moreover, the direct and indirect coupling terms can interfere constructively or destructively, facilitating or inhibiting the PCEnT process. We provide a general algorithm and procedure to calculate the input quantities for this vibronic coupling expression and apply it to the PCEnT process occurring in an anthracene–phenol–pyridine triad. We find that the indirect coupling is comparable in magnitude to the direct coupling at most proton donor–acceptor distances. The theory and methods developed in this work will enable the calculation of vibronic couplings and therefore rate constants for a wide range of PCEnT processes.

## Graphical abstract

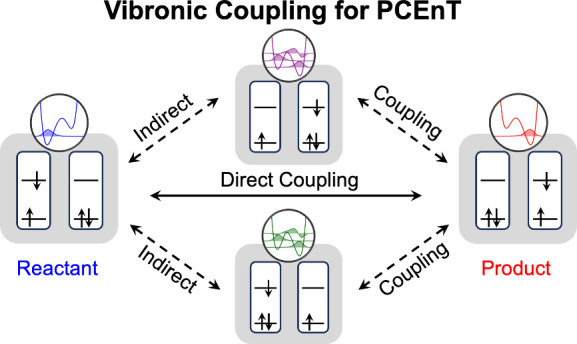

## 1 Introduction

In proton-coupled electron transfer reactions, the coupling between proton and electron transfer offers routes to control both components.^1–6^ Analogously, coupling proton transfer to excitation energy transfer (EnT) may also enable similar tunability. Previously, a photochemical process termed proton-coupled energy transfer (PCEnT) was predicted theoretically^7,8^ and discovered experimentally in the anthracene–phenol−pyridine (An-PhOH-py) triad system in a butyronitrile glass at 77 K, where photoexcitation of the An moiety leads to fluorescence of the PhOH-py in its keto form.^9^ This observation indicates that the electronic excitation energy is transferred from the An to the PhOH-py, coupled to proton transfer from phenol to pyridine. Without proton transfer, the excitation energy would not transfer because the energy of excited PhOH-py in the enol form is much higher than the energy of excited An. The proton transfer also would not occur without EnT because the enol tautomer of PhOH-py is more stable than the keto tautomer on the ground state. Recently, experiments have also shown that PCEnT occurs in the same or modified triad systems at room temperature^10,11^ and intermolecularly for different species at room temperature in solution.^12^

To understand the underlying physical principles of PCEnT, we developed an analytical theory for PCEnT and derived an expression for the nonadiabatic PCEnT rate constant.^13^ In this theory, the transferring proton and the electrons are treated quantum mechanically, and the PCEnT process is described in terms of nonadiabatic transitions between the reactant and product electron–proton vibronic states. For vibronically nonadiabatic PCEnT, the rate constant can be expressed as a summation over reactant and product vibronic states,

1 $$k_{\mathrm{PCEnT}}=\frac{1}{4\pi^{2}\hbar^{2}}\underset{\mu ,\nu }{\sum }P_{\mu }|V_{\mu \nu }{|}^{2}I_{\mu \nu }$$

Here, *P*_μ_ is the Boltzmann population of the reactant vibronic state μ, *V*_*μν*_ is the vibronic coupling between the reactant and product diabatic vibronic states μ and ν, and *I*_*μν*_ is the spectral convolution integral, which quantifies the probability that fluctuations of the solvent and/or solute lead to the crossing point between the reactant and product vibronic states. When the reorganization of common vibrational modes that couple to both the energy donor and acceptor is negligible during the PCEnT process, *I*_*μν*_ reduces to the spectral overlap integral between the donor emission and acceptor absorption line shape functions. Note that these line shape functions describe transitions between vibronic states rather than electronic states. Using this theory, we were able to explain the physical behavior of one of the triads and reproduce the experimental rate constant within an order of magnitude.^14^

A conventional EnT process may be categorized as occurring via the Förster mechanism, the Dexter mechanism, or both, based on whether the dipole–dipole interaction or short-range interaction dominates the electronic coupling.^15^ Experimentally, the Förster and Dexter mechanisms can be distinguished by measuring the dependence of the EnT rate constant on the donor–acceptor distance, whereas theoretically, they can be distinguished through a decomposition analysis of the electronic coupling.^16^ Intuitively, the same categorization should be applicable to PCEnT processes by analyzing the contributions of dipole–dipole and short-range interactions to the total vibronic coupling *V*_*μν*_. In addition, for conventional EnT, often higher-order perturbation terms must be included to correctly describe the Dexter mechanism.^17–20^ These higher-order terms represent virtual state-mediated interactions between the reactant and product states, which are also short-range interactions. However, the formulation of higher-order vibronic coupling terms has not been developed for PCEnT.

The goal of this paper is to derive a general expression for the vibronic coupling of PCEnT that encompasses both Förster and Dexter mechanisms using time-dependent perturbation theory. For PCEnT, the virtual states involved in the higher-order coupling terms will be electron–proton vibronic states instead of electronic states. Using this general expression, we will identify the dominant mechanism for PCEnT in the triad system. This PCEnT is a singlet–singlet process, indicating that both Förster and Dexter mechanisms could be operating. Both the An and PhOH-py moieties have large enough transition dipole moments to yield a significant dipole–dipole interaction, favoring the Förster mechanism. Thus, in our previous work, we assumed the Förster mechanism and represented the vibronic coupling by only the dipole–dipole term. However, due to the short distance between the An and PhOH-py moieties in the triad (5.92 Å),^14^ the Dexter mechanism could also take place, and the virtual vibronic states could also enable indirect coupling. Therefore, a quantitative analysis is needed to fully understand the PCEnT mechanism in the context of the vibronic coupling for this system.

This paper is organized as follows. In Section 2, we briefly review the general expression for electronic coupling in conventional EnT and show how this general expression can be reduced to the familiar expressions for Förster and Dexter EnT. In Section 3, we derive an analytical expression for the vibronic coupling for PCEnT processes. We show that this vibronic coupling consists of direct Coulomb and exchange terms, as well as indirect coupling terms, as in conventional EnT. Practical methods that can be used to obtain the input quantities to calculate the vibronic coupling within this formulation are also discussed. In Section 4, we apply this analysis to one of the An-PhOH-py triad systems. We quantify the contributions from direct and indirect coupling to the total vibronic coupling and assess the validity of the dipole–dipole approximation for this system. Finally, we discuss scenarios for which the indirect coupling must be included in a vibronic coupling calculation.

## 2 Theory

### 2.1 Electronic Coupling in Conventional Energy Transfer

In a conventional EnT process,

2 $$D^{*}A\to {\mathrm{DA}}^{*}$$

the electronic excitation energy is transferred from the energy donor (D) to the acceptor (A). Here * indicates that the donor or the acceptor is in the excited electronic state. Both direct and charge-transfer (CT) mediated interactions contribute to the coupling between the reactant state |D*A⟩ and the product state |DA*⟩.^17–19,21,22^ Figure 1 shows the interaction pathways linking |D*A⟩ and |DA*⟩ for a singlet–singlet EnT process. The horizontal line represents the direct interaction, whereas the angled edges represent the indirect interactions mediated by the CT states, |D^+^A^–^⟩ and |D^–^A^+^⟩.

**Figure 1:**
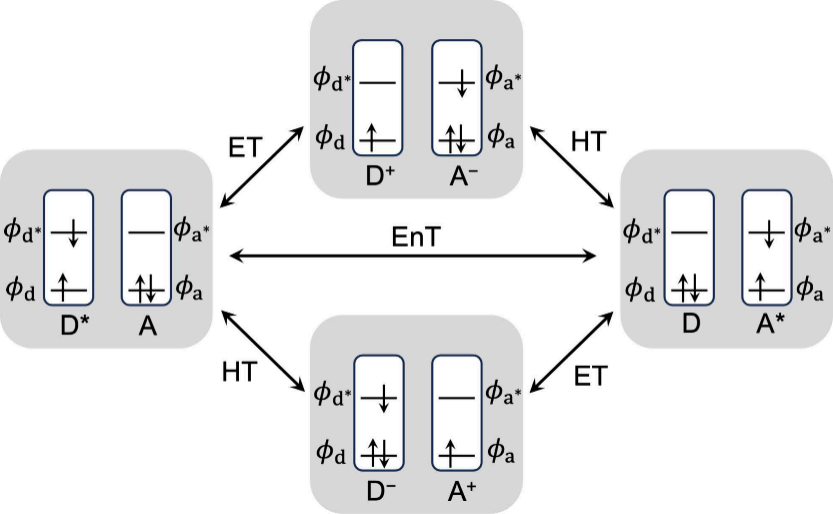
Possible direct and indirect coupling pathways for conventional singlet–singlet EnT. ET and HT denote electron transfer and hole transfer, respectively.

A general expression for the electronic coupling associated with this EnT process is given by

3 $$V_{\mathrm{el}}=V^{\left(1\right)}+V^{\left(2\right)}$$

where

4 $$V^{\left(1\right)}=\left\langle D^{*}A\right|{Ĥ}_{\mathrm{el}}\left|{\mathrm{DA}}^{*}\right\rangle$$

is the direct coupling term, ${Ĥ}_{\mathrm{el}}$ is the electronic Hamiltonian of the system, and

5 $$V^{\left(2\right)}=\frac{\left\langle D^{*}A\right|{Ĥ}_{\mathrm{el}}\left|D^{+}A^{-}\right\rangle \left\langle D^{+}A^{-}\right|{Ĥ}_{\mathrm{el}}\left|{\mathrm{DA}}^{*}\right\rangle }{E_{{\mathrm{DA}}^{*}}-E_{D^{+}A^{-}}}+\frac{\left\langle D^{*}A\right|{Ĥ}_{\mathrm{el}}\left|D^{-}A^{+}\right\rangle \left\langle D^{-}A^{+}\right|{Ĥ}_{\mathrm{el}}\left|{\mathrm{DA}}^{*}\right\rangle }{E_{{\mathrm{DA}}^{*}}-E_{D^{-}A^{+}}}$$

is the CT-mediated indirect coupling term,^18,21,22^ which can be derived from second-order time-dependent perturbation theory.^23^ This term is indirect because the interaction between states |D*A⟩ and |DA*⟩ occurs through their interaction with a virtual intermediate state |D^+^A^–^⟩ or |D^–^A^+^⟩. Note that the expression for the indirect coupling does not imply a sequential electron transfer–hole transfer or hole transfer–electron transfer mechanism. These CT states are not real intermediates of the energy transfer process but rather are only virtual states. According to the golden rule formalism, the rate constant for a transition is nonzero only when the energy is conserved. In this case, the most relevant geometries are the crossing points of the energy surfaces corresponding to the states |D*A⟩ and |DA*⟩ along the nuclear coordinates. At these geometries, microscopic reversibility is satisfied because |*V*_el_| is the same for the forward and backward processes.

The direct coupling term consists of Coulomb and short-range contributions.^16,24,25^ For the simplest case, consider the model EnT system shown in Figure 1, where ϕ_d_ and ϕ_a_ denote the highest occupied molecular orbital (HOMO), and $\phi_{d^{*}}$ and $\phi_{a^{*}}$ denote the lowest unoccupied molecular orbital (LUMO) of the donor (d) and acceptor (a). The excited states of both the donor and the acceptor are assumed to be dominated by the HOMO → LUMO transition. In this case, the diabatic states |D*A⟩ and |DA*⟩ are given by the determinants

6 $$\begin{array}{ll} & \left|D^{*}A\right\rangle =\left|\phi_{d}{\mathrm{\phi ̅}}_{d^{*}}\phi_{a}{\mathrm{\phi ̅}}_{a}\right\rangle \\ & \left|{\mathrm{DA}}^{*}\right\rangle =\left|\phi_{d}{\mathrm{\phi ̅}}_{d}\phi_{a}{\mathrm{\phi ̅}}_{a^{*}}\right\rangle \end{array}$$

where the orbitals with and without a bar indicate different electronic spins. The coupling between these determinants is

7 $$\left\langle D^{*}A\right|{Ĥ}_{\mathrm{el}}\left|{\mathrm{DA}}^{*}\right\rangle =\left(d^{*}d|{\mathrm{aa}}^{*}\right)-\left(d^{*}a^{*}|\mathrm{ad}\right)\equiv J-K$$

where

8 $$\begin{array}{ll} J & =\left(d^{*}d|{\mathrm{aa}}^{*}\right)=\iint \phi_{d^{*}}\left(r_{1}\right)\phi_{d}\left(r_{1}\right)\frac{1}{r_{12}}\phi_{a}\left(r_{2}\right)\phi_{a^{*}}\left(r_{2}\right)dr_{1}dr_{2}\\ K & =\left(d^{*}a^{*}|\mathrm{ad}\right)=\iint \phi_{d^{*}}\left(r_{1}\right)\phi_{a^{*}}\left(r_{1}\right)\frac{1}{r_{12}}\phi_{a}\left(r_{2}\right)\phi_{d}\left(r_{2}\right)dr_{1}dr_{2} \end{array}$$

are the Coulomb and exchange integrals, respectively, and *r*_12_ = | ***r***_1_ – ***r***_2_|. We have assumed that the orbitals ϕ_d_, ϕ_a_, $\phi_{d^{*}}$ , and $\phi_{a^{*}}$ are real.

For Förster energy transfer, usually the separation between the energy donor and acceptor is larger than the size of the molecules, and a multipole expansion is applicable to the Coulomb integral *J*.^26,27^ The leading term is the dipole–dipole interaction, which is given by (in atomic units)

9 $$J\approx J_{\mathrm{dipole}}=\kappa \frac{\left|d_{D}∥d_{A}\right|}{R^{3}}$$

where κ is the orientation factor, ***d***_D_ and ***d***_A_ are the transition dipole moments for the energy donor and acceptor, and *R* is the distance between the energy donor and acceptor. On the other hand, both the exchange interaction and the indirect interaction depend on the overlap between the molecular orbitals of the donor and the acceptor and therefore decay exponentially with the intermolecular distance *R*. These terms become negligible at large energy donor–acceptor separation. In this case, the total electronic coupling can be approximated as^27–29^

10 $$V_{\mathrm{el}}^{\mathrm{Förster}}\approx J_{\mathrm{dipole}}$$

For Dexter-type triplet–triplet EnT, the diabatic states |^3^D*A⟩ and |D^3^A*⟩ are

11 $$\begin{array}{ll} & {|}^{3}D^{*}A\rangle =\left|\phi_{d}\phi_{d^{*}}\phi_{a}{\mathrm{\phi ̅}}_{a}\right\rangle \\ & \left|D^{3}A^{*}\right\rangle =\left|\phi_{d}{\mathrm{\phi ̅}}_{d}\phi_{a}\phi_{a^{*}}\right\rangle \end{array}$$

and the direct coupling between these determinants is

12 V(1)=⟨D*3A|Ĥel|D3A*⟩=−(d*a*|ad)≡−K

The Coulomb integral *J* becomes zero because of the orthogonality between electronic spin orbitals with different spin. In this case,

13 $$V_{\mathrm{el}}^{\mathrm{Dexter}}\approx -K+V^{\left(2\right)}$$

Note that although *V* ^(2)^ is a higher-order term, its magnitude may be comparable with or even larger than the first-order term *K*, and thus it must be included to accurately calculate the electronic coupling for the Dexter mechanism.^17,19^ In general, for singlet–singlet EnT with a shorter energy donor–acceptor distance, both Förster and Dexter mechanisms may be operating, and the dominant mechanism is determined by the relative magnitudes of the *J*, *K*, and *V* ^(2)^ terms. For Dexter-type singlet–singlet EnT, −*K* + *V* ^(2)^ will be dominant.

### 2.2 Vibronic Coupling in PCEnT

Analogous to the electronic coupling in conventional EnT, a similar treatment can be applied to the vibronic coupling in PCEnT. In nonadiabatic PCEnT theory,^13^ the transferring proton as well as the electrons are treated quantum mechanically. The Hamiltonian of the electron–proton subsystem is

14 $${Ĥ}_{\mathrm{ep}}={\mathrm{T̂}}_{p}+{\mathrm{T̂}}_{e}+V\left(r_{e},r_{p},q\right)\equiv {\mathrm{T̂}}_{p}+{Ĥ}_{\mathrm{el}}$$

where ${\mathrm{T̂}}_{p}$ , ${\mathrm{T̂}}_{e}$ , and *V* are the kinetic energy operator for the transferring proton, the kinetic energy operator for the electrons, and the potential energy, respectively. Here, ***r***_e_, ***r***_p_, and ***q*** are the coordinates of the electrons, the transferring proton, and the bath (i.e., the solvent and solute modes), respectively. An electron–proton vibronic state of the subsystem is denoted as |*j*ξ⟩, where *j* indicates the electronic diabatic state and ξ indicates a proton vibrational state associated with the electronic state |*j*⟩. Following the notation of previous work,^13,14^ we use |Iμ⟩ and |IIν⟩ to denote the reactant and product vibronic states, respectively.

The electronic diabatic state |*j*⟩ should satisfy the condition that the derivative coupling with respect to the proton coordinate vanishes,^30^

15 $$\langle j\left|\nabla_{r_{p}}\right|k\rangle =0,\mathrm{for all}j,k$$

Here, $\nabla_{r_{p}}$ denotes the gradient with respect to the proton coordinate. For multidimensional systems, the condition in eq 15 can typically only be approximately satisfied.^31^ In practice, the diabatic states are often constructed using techniques such as application of charge or spin constraints or rotation of the adiabatic states, and we assume that the condition in eq 15 is approximately satisfied.

The proton vibrational states |ξ^(*j*)^⟩ associated with electronic diabatic state |*j*⟩ can be obtained from

16 $$\left[{\mathrm{T̂}}_{p}+E_{j}\left(r_{p},q\right)\right]\left|\xi^{\left(j\right)}\right\rangle =E_{j\xi }\left(q\right)\left|\xi^{\left(j\right)}\right\rangle$$

Here, $E_{j}\left(r_{p},q\right)\equiv \left\langle j\right|{Ĥ}_{\mathrm{el}}\left|j\right\rangle$ is the electronic energy of the diabatic state |*j*⟩ at the proton coordinate ***r***_p_ and bath coordinate ***q***, and *E*_*jξ*_(***q***) is the energy of the diabatic vibronic state |*jξ*⟩ at the bath coordinate ***q***. The superscript (*j*) on the proton vibrational states will only be used when the corresponding electronic state could be ambiguous; otherwise the superscript will be suppressed for notational simplicity. Note that |*jξ*⟩ is not an eigenstate of ${Ĥ}_{\mathrm{ep}}$ .

The direct coupling between the reactant vibronic state |Iμ⟩ and the product vibronic state |IIν⟩ is

17 $$V_{\mu \nu }^{\left(1\right)}=\left\langle I\mu \right|{Ĥ}_{\mathrm{ep}}\left|\mathrm{II}\nu \right\rangle$$

For notational simplicity, the subscripts μ and ν in *V*_*μν*_ will always correspond to electronic states |I⟩ and |II⟩. The second-order, indirect coupling for PCEnT can be written analogously to eq 5, but we need to sum over all possible vibronic states, instead of just the two CT electronic states:

18 $$V_{\mu \nu }^{\left(2\right)}=\underset{j}{\sum }\underset{\xi }{\sum }\frac{\left\langle I\mu \right|{Ĥ}_{\mathrm{ep}}\left|j\xi \right\rangle \left\langle j\xi \right|{Ĥ}_{\mathrm{ep}}\left|\mathrm{II}\nu \right\rangle }{E_{\mathrm{II}\nu }-E_{j\xi }}$$

The terms in this expression depend on the bath coordinate ***q***. In the regime where the golden rule formalism is valid, the rate constant for a transition is nonzero only when the energy is conserved. In this context, the most relevant geometries are the crossing points of the energy surfaces corresponding to the states |Iμ⟩ and |IIν⟩ along the bath coordinates. At these geometries, microscopic reversibility is satisfied because |*V*_*μν*_| = |*V*_*νμ*_|.

The virtual intermediate states |*jξ*⟩ can be categorized into two sets:

1. *j* = I, ξ ≠ μ or *j* = II, ξ ≠ ν. The virtual intermediate state is a different proton vibrational state associated with the same electronic state as the reactant or product. The matrix element $\left\langle I\mu \right|{Ĥ}_{\mathrm{ep}}\left|I\xi \right\rangle$ and $\left\langle \mathrm{II}\xi \right|{Ĥ}_{\mathrm{ep}}\left|\mathrm{II}\nu \right\rangle$ will be zero because the derivative coupling between the diabatic states is assumed to vanish and the proton vibrational wave functions associated with the same electronic state are orthogonal. Thus, these virtual intermediate states do not contribute to $V_{\mu \nu }^{\left(2\right)}$ .
2. *j* ≠ I or II. For this case, the electronic diabatic state |*j*⟩ most likely has CT character. This scenario corresponds to indirect interaction pathways through a virtual proton-coupled CT intermediate state, as illustrated in Figure 2. All proton vibrational states associated with the virtual electronic state contribute to the indirect coupling.

**Figure 2:**
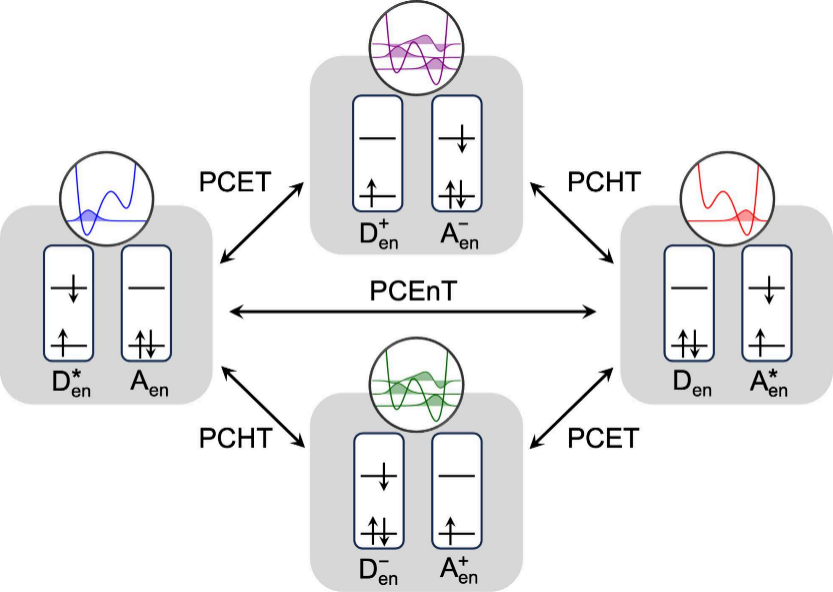
Possible direct and indirect PCEnT coupling pathways between the vibronic states |I0⟩ and |II0⟩. The circled plots depict the proton potential energy profiles and corresponding proton vibrational wave functions associated with each electronic diabatic state. For the two virtual CT states, all proton vibrational states contribute to the indirect coupling according to eq 18. PCET and PCHT denote proton-coupled electron transfer and proton-coupled hole transfer, respectively.

The third-order coupling, which describes the indirect interaction between the reactant and product vibronic states through two virtual intermediate states, can be expressed as

19 $$V_{\mu \nu }^{\left(3\right)}=\underset{j,k}{\sum }\underset{\xi ,\eta }{\sum }\frac{\left\langle I\mu \right|{Ĥ}_{\mathrm{ep}}\left|j\xi \right\rangle \left\langle j\xi \right|{Ĥ}_{\mathrm{ep}}\left|k\eta \right\rangle \left\langle k\eta \right|{Ĥ}_{\mathrm{ep}}\left|\mathrm{II}\nu \right\rangle }{\left(E_{\mathrm{II}\nu }-E_{j\xi }\right)\left(E_{\mathrm{II}\nu }-E_{k\eta }\right)}$$

With a similar argument as above, only the virtual states that satisfy *j* ≠ I, *k* ≠ II, and *j* ≠ *k* contribute to $V_{\mu \nu }^{\left(3\right)}$ . This higher-order coupling is expected to be small and will not be considered herein. One exception is when the EnT is mediated by a bridge connecting the energy donor and acceptor. In this case, the third- and even fourth-order terms could be important.^18,19^

The total PCEnT vibronic coupling between the reactant vibronic state |Iμ⟩ and the product vibronic state |IIν⟩ is the sum of the first-order and second-order terms,

20 $$V_{\mu \nu }=V_{\mu \nu }^{\left(1\right)}+V_{\mu \nu }^{\left(2\right)}$$

Since the PCEnT rate constant is proportional to $\left|V_{\mu \nu }{|}^{2}=\right|V_{\mu \nu }^{\left(1\right)}+V_{\mu \nu }^{\left(2\right)}{|}^{2}\neq \left|V_{\mu \nu }^{\left(1\right)}{|}^{2}+\right|V_{\mu \nu }^{\left(2\right)}{|}^{2}$ , quantum interference^19^ between $V_{\mu \nu }^{\left(1\right)}$ and $V_{\mu \nu }^{\left(2\right)}$ is expected.

The matrix elements of ${Ĥ}_{\mathrm{ep}}$ , $\left\langle j\xi \right|{Ĥ}_{\mathrm{ep}}\left|k\eta \right\rangle$ , can be calculated using a semiclassical formulation that expresses this matrix element as the product of a prefactor κ and the adiabatic vibronic coupling, where κ depends on the properties of the diabatic proton potential energy curves as well as the electronic coupling.^32,33^ This general expression spans the electronically adiabatic and nonadiabatic limits as well as the intermediate regime. In the electronically nonadiabatic limit, κ < 1, and the matrix elements of ${Ĥ}_{\mathrm{ep}}$ are approximately

21 $$\left\langle j\xi \right|{Ĥ}_{\mathrm{ep}}\left|k\eta \right\rangle \approx \left\langle j\right|{Ĥ}_{\mathrm{el}}\left|k\right\rangle \left\langle \xi^{\left(j\right)}\right|\eta^{\left(k\right)}\rangle \equiv \left\langle j\right|{Ĥ}_{\mathrm{el}}\left|k\right\rangle S_{j\xi ,k\eta }$$

where *S*_*jξ*,*kη*_ = ⟨ξ^(*j*)^|η^(*k*)^⟩ is the overlap integral between proton vibrational wave functions associated with electronic diabatic states *j* and *k*. Here, the electronic coupling is assumed to be independent of the proton coordinate, although this assumption is not essential and could be removed by numerical integration of the product of the electronic coupling and the proton vibrational wave functions ξ^(*j*)^ and η^(*k*)^ over the proton coordinate.^34^ The final expression for the vibronic coupling of a PCEnT reaction in the electronically nonadiabatic limit becomes

22 $$V_{\mu \nu }=\left\langle I\right|{Ĥ}_{\mathrm{el}}\left|\mathrm{II}\right\rangle S_{I\mu ,\mathrm{II}\nu }+\underset{j\neq \mathrm{I,II}}{\sum }\underset{\xi }{\sum }\frac{\left\langle I\right|{Ĥ}_{\mathrm{el}}\left|j\right\rangle \left\langle j\right|{Ĥ}_{\mathrm{el}}\left|\mathrm{II}\right\rangle }{E_{\mathrm{II}\nu }-E_{j\xi }}S_{I\mu ,j\xi }S_{j\xi ,\mathrm{II}\nu }$$

In this expression for the total vibronic coupling, the first term, $V_{\mu \nu }^{\left(1\right)}$ , is the direct vibronic coupling, and the second term, $V_{\mu \nu }^{\left(2\right)}$ , is the indirect vibronic coupling.

In the electronically nonadiabatic limit, we can apply similar approximations for the electronic coupling terms as in conventional EnT. At large energy donor–acceptor distance *R*, $\left\langle I\right|{Ĥ}_{\mathrm{el}}\left|\mathrm{II}\right\rangle \approx J_{\mathrm{dipole}}$ , and $\left\langle I\right|{Ĥ}_{\mathrm{el}}\left|j\right\rangle$ and $\left\langle j\right|{Ĥ}_{\mathrm{el}}\left|\mathrm{II}\right\rangle$ are negligible for cases where the intermediate states are associated with electron or hole transfer because they decay exponentially with *R*. In such cases, the vibronic coupling can be approximated as in our previous work,^14^

23 $$V_{\mu \nu }^{\mathrm{Förster}}\approx J_{\mathrm{dipole}}S_{I\mu ,\mathrm{II}\nu }$$

For a triplet–triplet PCEnT process, $\left\langle I\right|{Ĥ}_{\mathrm{el}}\left|\mathrm{II}\right\rangle =-K$ , and

24 $$V_{\mu \nu }^{\mathrm{Dexter}}=-KS_{I\mu ,\mathrm{II}\nu }+V_{\mu \nu }^{\left(2\right)}$$

Similar to conventional EnT, for singlet–singlet PCEnT with a relatively short energy donor–acceptor distance, both Förster and Dexter mechanisms may be operating, and the dominant mechanism is determined by the relative magnitudes of the *JS*_Iμ,IIν_, *KS*_Iμ,IIν_, and $V_{\mu \nu }^{\left(2\right)}$ terms. For Dexter-type singlet–singlet PCEnT, $-KS_{I\mu ,\mathrm{II}\nu }+V_{\mu \nu }^{\left(2\right)}$ will be dominant.

We summarize the assumptions used in the derivation of the general expression for the vibronic coupling here. The vibronic coupling is a parameter in the PCEnT rate constant expression in eq 1, which is valid only for incoherent PCEnT in the vibronically nonadiabatic limit. We assumed that the electronic diabatic states can be constructed such that the nonadiabatic coupling between them approximately vanishes. The diabatic vibronic states are then computed as the product of an electronic diabatic state and an associated proton vibrational state. In addition, the matrix elements of ${Ĥ}_{\mathrm{ep}}$ , $\left\langle j\xi \right|{Ĥ}_{\mathrm{ep}}\left|k\eta \right\rangle$ , were assumed to be independent of the bath coordinate, except for the proton donor–acceptor distance, which is treated explicitly in PCEnT theory by thermally averaging the rate constant over this distance. Lastly, the electronically nonadiabatic limit and the weak dependence of the electronic coupling on the proton coordinate are assumed for eqs 22–24. However, the general expressions in eqs 17–20 do not rely on these assumptions.

## 3 Computational Methods

### 3.1 Electronic Structure Calculations

The electronic structure calculations were performed with the Q-Chem 6 package^35^ using time-dependent density functional theory (TDDFT) with the Tamm-Dancoff approximation (TDA).^36^ The 6-31+G­(d,p) basis set^37–39^ was used in conjunction with two different functionals, CAM-B3LYP^40^ and ωB97XD.^41^ These two functionals produced qualitatively similar results. All single-point energy calculations and geometry optimizations were performed in the gas phase.

### 3.2 Minimum Energy Crossing Point Optimization

In the context of the golden rule rate constant, the vibronic couplings *V*_*μν*_ at the crossing points of the energy surfaces corresponding to the states |Iμ⟩ and |IIν⟩ along the bath coordinates are the most relevant. In practice, we calculate the vibronic coupling at geometries such that *E*_Iμ_ = *E*_IIν_. Moreover, the PCEnT reaction is expected to occur near the minimum energy crossing point (MECP), at which *E*_Iμ_ and *E*_IIν_ are minimized with respect to the bath coordinate ***q***. This assumption is valid at low temperature, where the thermal fluctuations of the bath are limited. At higher temperature, especially in the presence of low-frequency bath modes that strongly affect the coupling (e.g., the twist of the dihedral angle between phenol and pyridine), we will need to generate an ensemble of crossing point geometries, calculate the vibronic coupling at each geometry, and thermally average the resulting rate constant over the relevant modes, in addition to thermal averaging over the proton donor–acceptor distance. In this work, we focus on calculating the vibronic coupling at the MECP geometry.

The constraint *E*_Iμ_(***q*** *) = *E*_IIν_(***q*** *), where ***q*** * is the MECP geometry, can be rewritten as

25 $$E_{I}\left(r_{\mathrm{p,eq}}^{\left(I\right)},q^{*}\right)+\varepsilon_{\mu }^{\left(I\right)}=E_{\mathrm{II}}\left(r_{\mathrm{p,eq}}^{\left(\mathrm{II}\right)},q^{*}\right)+\varepsilon_{\nu }^{\left(\mathrm{II}\right)}$$

Here, *E*_I/II_ is the energy of the reactant/product electronic diabatic state, $r_{\mathrm{p,eq}}^{\left(I/\mathrm{II}\right)}$ is the equilibrium position of the transferring proton in the reactant/product state, and $\varepsilon_{\mu }^{\left(I\right)}$ and $\varepsilon_{\nu }^{\left(\mathrm{II}\right)}$ are the reactant and product proton vibrational energy levels relative to the minimum of the respective proton potential energy profile. This treatment assumes that the proton vibrational energy levels do not strongly depend on the bath coordinate.

In practice, $r_{\mathrm{p,eq}}^{\left(I\right)}$ , $r_{\mathrm{p,eq}}^{\left(\mathrm{II}\right)}$ , and ***q*** * are all unknown and are obtained through geometry optimization. Note that the constraint given in eq 25 should only affect the optimization with respect to ***q***, whereas the transferring proton should be optimized freely. The constrained optimization can be achieved by minimizing the following objective function with respect to ***q***,

26 $$J\left(r_{p1},r_{p2},q\right)=\frac{E_{I}\left(r_{p1},q\right)+E_{\mathrm{II}}\left(r_{p2},q\right)+\varepsilon_{\mu }^{\left(I\right)}+\varepsilon_{\nu }^{\left(\mathrm{II}\right)}}{2}+c{\left[E_{I}\left(r_{p1},q\right)-E_{\mathrm{II}}\left(r_{p2},q\right)-\Delta_{\mu \nu }\right]}^{2}$$

where ***r***_p1_, ***r***_p2_, and ***q*** are independent variables, *c* is a positive constant, and $\Delta_{\mu \nu }=\varepsilon_{\nu }^{\left(\mathrm{II}\right)}-\varepsilon_{\mu }^{\left(I\right)}$ . The second term in J is the penalty term, which causes J to increase if the system deviates from the constraint. The constant *c* controls the strength of the penalty. We also minimize *E*_I_(***r***_p1_, ***q***) and *E*_II_(***r***_p2_, ***q***) with respect to ***r***_p1_ and ***r***_p2_ at a given ***q***, respectively, to obtain $r_{\mathrm{p,eq}}^{\left(I\right)}$ and $r_{\mathrm{p,eq}}^{\left(\mathrm{II}\right)}$ . The overall optimization process can be summarized as

27 $${\underset{r_{p1}}{\min}\;E_{I}\left(r_{p1},q\right)|}_{\mathrm{fix}\;q},{\underset{r_{p2}}{\min}\;E_{\mathrm{II}}\left(r_{p2},q\right)|}_{\mathrm{fix}\;q},{\underset{q}{\min}\;J\left(r_{p1},r_{p2},q\right)|}_{\mathrm{fix}\;r_{p1},r_{p2}}$$

We implement the MECP optimization procedure as follows. First, we create two copies of the system, whose coordinates are {***r***_p1_, ***q***} and {***r***_p2_, ***q***}, respectively. The coordinates of nuclei other than the transferring proton are the same in the two systems. We then perform electronic structure calculations to obtain *E*_I_(***r***_p1_, ***q***), *E*_II_(***r***_p2_, ***q***), and the gradients $\frac{\partial E_{I}}{\partial r_{p1}}$ , $\frac{\partial E_{I}}{\partial q}$ , $\frac{\partial E_{\mathrm{II}}}{\partial r_{p2}}$ , and $\frac{\partial E_{\mathrm{II}}}{\partial q}$ . Note that *E*_I_ and *E*_II_ are computed as the adiabatic energies when the proton is localized near its donor or acceptor, respectively, because the adiabatic and diabatic states are expected to be essentially equivalent at these geometries. $J\left(r_{p1},r_{p2},q\right)$ can then be calculated using eq 26, and its gradient with respect to ***q*** is

28 $$\frac{\partial J}{\partial q}=\frac{1}{2}\left(\frac{\partial E_{I}}{\partial q}+\frac{\partial E_{\mathrm{II}}}{\partial q}\right)+2c\left(E_{I}-E_{\mathrm{II}}-\Delta_{\mu \nu }\right)\left(\frac{\partial E_{I}}{\partial q}-\frac{\partial E_{\mathrm{II}}}{\partial q}\right)$$

We perform the three optimizations in eq 27 to obtain the updated coordinates {***r***_p1_, ***r***_p2_, ***q***} and the updated *E*_I_, *E*_II_, and J . The above steps are repeated until the forces on the atoms and the absolute energy deviation, |*E*_I_ – *E*_II_ – Δ_*μν*_|, are smaller than specified thresholds.

For the triad system, we focus on the coupling between the |I0⟩ and |II0⟩ vibronic states and set Δ_00_ = 0, neglecting the difference in the zero-point energies when computing the MECP. We initially set *c* = 25 eV^–1^ and increased it as needed to ensure the final absolute energy deviation |*E*_I_ – *E*_II_| < 0.01 eV after optimization. The atomic coordinates were updated using the limited-memory Broyden–Fletcher–Goldfarb–Shanno (LBFGS) algorithm.^42^ The geometry optimization was considered to be converged when the forces on all atoms were less than 0.03 eV/Å.

### 3.3 Proton Vibrational States

We generated the proton potential energy profiles for the relevant electronic diabatic states (i.e., |I⟩, |II⟩, and the intermediate states) at the MECP geometry by moving the proton along a grid spanning the proton axis and performing single-point energy calculations. The proton axis is defined as the line connecting $r_{\mathrm{p,eq}}^{\left(I\right)}$ and $r_{p,eq}^{\left(\mathrm{II}\right)}$ at the MECP geometry. The proton vibrational energy levels and wave functions were then obtained by solving the one-dimensional Schrödinger equation for the proton potential energy profile associated with each electronic diabatic state using the Fourier grid Hamiltonian method.^43^ The overlap integrals between the proton vibrational wave functions were calculated numerically.

### 3.4 Electronic Coupling Calculation

After generating the proton potential energy profiles along the one-dimensional proton coordinate, we identified the crossing point at which the proton potential curves for the reactant and product states intersect. The electronic coupling was calculated at the geometry where the transferring proton is located at this crossing point. This choice is reasonable because the electronic couplings do not depend strongly on *r*_p_ (Figure S4), and the diabatization methods described below work best when the states of interest are well-separated from other excited states.

When calculating the electronic coupling between the local excited state (LES) of the anthracene and the local electron–proton transfer (LEPT) state associated with the phenol-pyridine of the triad, constrained density functional theory-based methods^44^ are not applicable. We used an eigenstate-based method, where the diabatic states can be constructed from adiabatic states using Edmiston-Ruedenberg (ER) or occupied-virtual separated Boys (BoysOV) diabatization.^16,45–47^ These methods can simultaneously diabatize more than two excited states and enable the calculation of the electronic coupling between any pair of the resulting diabatic states. If {|Ψ_*i*_⟩} denotes the set of adiabatic states obtained from an excited state calculation using a method such as TDA-TDDFT, the diabatic states |Ξ_*j*_⟩ can be constructed via a unitary transformation,

29 $$\left|\Xi_{j}\right\rangle =\underset{i}{\sum }\left|\Psi_{i}\right\rangle U_{ij}$$

where **U** is the rotation matrix. **U** is determined by either ER diabatization^46^ or BoysOV diabatization.^45,47^ The diabatic electronic couplings are the off-diagonal terms in the transformed Hamiltonian,

30 $$\left\langle \Xi_{i}\right|{Ĥ}_{\mathrm{el}}\left|\Xi_{j}\right\rangle =\underset{k,l}{\sum }U_{ki}\left\langle \Psi_{k}\right|{Ĥ}_{\mathrm{el}}\left|\Psi_{l}\right\rangle U_{lj}$$

Since the ground state is not affected by this unitary transformation, we can calculate the transition dipole moments of the diabatic states as

31 $$d_{\Xi_{j}}=\left\langle \Psi_{G}\right|\mathrm{\mu ̂}\left|\Xi_{j}\right\rangle =\underset{i}{\sum }\left\langle \Psi_{G}\right|\mathrm{\mu ̂}\left|\Psi_{i}\right\rangle U_{ij}$$

where |Ψ_G_⟩ denotes the ground state and μ̂ is the dipole operator. $\left\langle \Psi_{G}\right|\mathrm{\mu ̂}\left|\Psi_{i}\right\rangle$ is the transition dipole moment of the adiabatic state |Ψ_*i*_⟩ and is computed during standard TDA-TDDFT calculations. The dipole–dipole coupling between the diabatic states can then be calculated using eq 9.

## 4 Results and Discussion

To demonstrate a practical application of this theoretical formulation, we calculated the vibronic coupling for the PCEnT process occurring in the An-PhOH-py triad system shown in Figure 3. In the triad system, the reactant state |I⟩ is the LES of the anthracene, and the product state |II⟩ is the LEPT state associated with the phenol-pyridine. According to eq 1, the pairs of vibronic states that dominate the PCEnT process are determined by a balance among the quantities *P*_μ_, *V*_*μν*_, and *I*_*μν*_. Our previous work showed that the (I0, II0) pair of vibronic states dominates the PCEnT process at 77 K, contributing more than 96% of the PCEnT rate constant, due to the much larger spectral overlap for this pair of vibronic states compared to all other pairs.^14^ Therefore, we focus on calculating the vibronic coupling between the |I0⟩ and |II0⟩ vibronic states.

**Figure 3:**
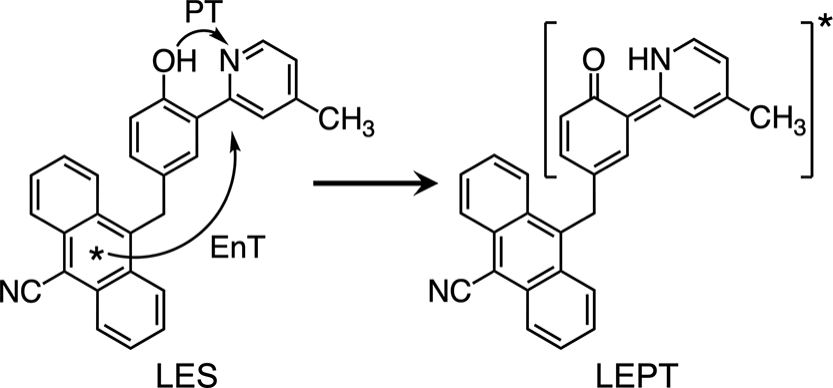
PCEnT process occurring in the An-PhOH-py triad system, where the transfer of electronic excitation energy from An to PhOH-py is coupled to a proton transfer reaction from PhOH to py.

### 4.1 Identifying Possible Virtual Intermediate States

First we need to identify the possible virtual intermediate states |*j*⟩ that can mediate the indirect interaction between the reactant and product vibronic states. For this initial analysis, we optimized the MECP geometry between the |I0⟩ and |II0⟩ vibronic states using the CAM-B3LYP functional by setting Δ_00_ = 0 in J and constraining the proton donor–acceptor distance (i.e., the distance between the phenol oxygen and the pyridine nitrogen atoms) to be 2.60 Å, which is approximately the equilibrium proton donor–acceptor distance. At the optimized MECP geometry, we generated the proton potential energy profiles for the relevant electronic states. The characters of the states were assigned by visually inspecting the dominant natural transition orbitals (NTOs),^48^ with confirmation from partial charge and transition dipole moment analyses. The LES, LEPT state, and charge separated state (CSS), where an electron is transferred from PhOH-py to An, are the lowest-lying excited states, consistent with previous work.^7–9,14^ In addition, three other low-lying excited states were identified, including one higher excited state with LES character (LES2), one higher excited state with LEPT character (LEPT2), and a state denoted as the “reverse charge separated state” (RCSS), where an electron is transferred from An to PhOH-py.

The proton potential energy profiles and associated proton vibrational wave functions associated with these electronic states are shown in Figure 4. All six electronic states exhibit proton potential energy profiles with asymmetric double-well character. For the LES and LES2, the lower well of the double-well potential is on the proton donor side, whereas for the other four states, the lower well of the double-well potential is on the proton acceptor side. The energies of the |I0⟩ and |II0⟩ vibronic states corresponding to the LES and LEPT state, respectively, align well (Figure 4a), with an energy difference of 0.007 eV, indicating the success of the MECP optimization algorithm.

**Figure 4:**
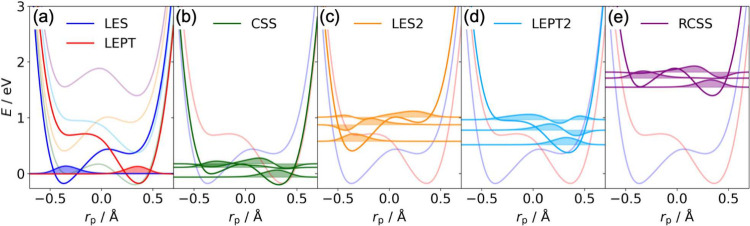
Proton potential energy profiles of the LES, LEPT state, CSS, LES2, LEPT2 state, and RCSS calculated at the MECP geometry corresponding to the LES and the LEPT state using the CAM-B3LYP functional. The ground proton vibrational wave functions associated with the LES and the LEPT state are shown in (a). The first three proton vibrational wave functions associated with the possible virtual intermediate states are shown in (b)–(e). Negative *r*_p_ indicates that the proton is closer to the proton donor (the O atom in PhOH), and positive *r*_p_ indicates that the proton is closer to the proton acceptor (the N atom in py). All energies were obtained directly from the TDA-TDDFT calculation and shifted by the same energy constant.

The first three proton vibrational wave functions associated with the CSS are shown in Figure 4b. At the MECP geometry for the |I0⟩ and |II0⟩ vibronic states, the CSS energies *E*_*jξ*_ are very close to the energy of the aligned reactant and product states, *E*_I0_ and *E*_II0_, shown in Figure 4a. Furthermore, since the proton potential energy barrier is low for the CSS, the proton vibrational wave functions associated with the CSS are relatively delocalized, resulting in large overlap with the reactant and product proton vibrational wave functions associated with the |I0⟩ and |II0⟩ states, respectively. According to eq 22, the indirect coupling is large if there exist some virtual vibronic states with energy *E*_*jξ*_ close to *E*_II0_ and significant proton vibrational wave function overlap with both the |I0⟩ and |II0⟩ vibronic states. We therefore expect a non-negligible contribution from the indirect interaction mediated by the CSS to the total vibronic coupling.

On the other hand, the LES2, LEPT2 state, and RCSS have much higher energies than the LES and LEPT state (Figure 4c–e). The vibronic energies of the LES2, LEPT2 state, and RCSS for their respective ground proton vibrational states are 0.58, 0.52, and 1.55 eV higher than *E*_I0_ and *E*_II0_, respectively. This large energy difference results in a large denominator in eq 22, which diminishes the contributions of the vibronic states associated with the LES2, LEPT2 state, and RCSS to the indirect vibronic coupling. Assuming all the electronic couplings between these electronic states are the same for this initial analysis, the vibronic couplings mediated by the LES2, LEPT2 state, and RCSS are 2 orders of magnitude smaller than the direct coupling and the indirect coupling mediated by the CSS (Table S1). Therefore, for the subsequent calculations we will only include the CSS as the virtual intermediate state |*j*⟩. Note that we will compute the electronic couplings explicitly in these subsequent calculations without assuming that any of them are the same.

### 4.2 Direct and Indirect Coupling at Various Proton Donor–Acceptor Distances

We calculated the vibronic coupling between the |I0⟩ and |II0⟩ vibronic states of the triad using the general expression in eq 22 at different proton donor–acceptor distances *R*_PT_ ranging from 2.45 Å to 2.80 Å with a spacing of 0.05 Å. The equilibrium proton donor–acceptor distance in this triad is around 2.60 Å, whereas the *R*_PT_ that dominates the PCEnT rate constant has been shown to be ∼2.50 Å.^14^ The shorter dominant distance is due to the thermal averaging of the PCEnT rate constant over *R*_PT_ because the rate constant increases dramatically as *R*_PT_ decreases. We optimized the MECP geometries between the LES and LEPT state, corresponding to the |I0⟩ and |II0⟩ vibronic states neglecting differences in zero-point energy, with constraints applied to *R*_PT_. The calculated proton potential energy profiles and the proton vibrational wave functions associated with the LES, LEPT state, and CSS at the MECP geometry at each *R*_PT_ value are shown in the Supporting Information. A representative MECP structure is shown in Figure S3. Note that the PhOH-py moiety has a planar geometry at the MECP, in contrast to the twisted PhOH-py moiety at the fully relaxed LEPT state equilibrium geometry. This finding suggests that the twist motion would occur after the crossing from the LES to the LEPT state and is not the driver of the PCEnT process.

The electronic couplings among the LES, LEPT state, and CSS were calculated with the transferring proton located at the point where the proton potential energy profiles for the LES and the LEPT state intersect. The ER diabatization method was used to construct the diabatic states from the singlet excited states S_1_, S_2_, and S_3_. BoysOV diabatization gives very similar results (see Supporting Information). For all *R*_PT_ values, the S_1_ state has CSS character, and the S_2_ and S_3_ states are superpositions of the LES and the LEPT state. The dominant NTOs of these states at a representative geometry are shown in Figure 5. The transition dipole moments of the diabatic states calculated using eq 31 were used to identify the LES and LEPT state after diabatization.

**Figure 5:**
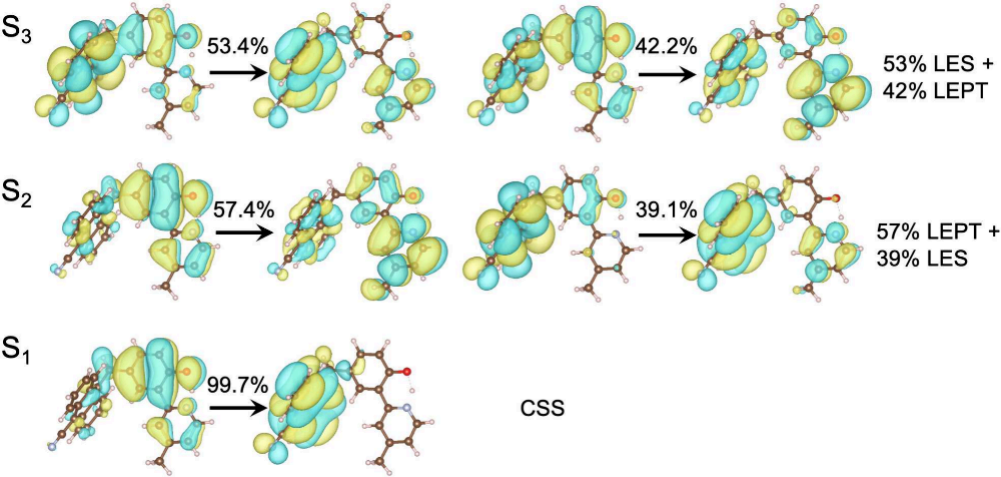
Dominant NTO pairs of the S_1_, S_2_, and S_3_ states calculated at the MECP geometry between the LES and the LEPT state with the transferring proton located at the point where the proton potential energy profiles intersect using the CAM-B3LYP functional at *R*_PT_ = 2.55 Å.

Figure 6 shows the electronic couplings among the LES, LEPT state, and CSS at various proton donor–acceptor distances *R*_PT_ calculated at the MECP geometry and proton potential crossing point corresponding to the LES and the LEPT state using two different functionals. The electronic couplings are qualitatively similar for the two functionals studied, in terms of the ordering for the three pairs of electronic diabatic states and the relatively weak dependence on the proton donor–acceptor distance *R*_PT_. The ωB97XD functional predicts similar but slightly larger magnitudes for the electronic couplings between the LES and LEPT state and between the LES and CSS compared to the CAM-B3LYP functional. The couplings involving the CSS obtained with the ωB97XD functional at *R*_PT_ ≤ 2.45 Å are less reliable due to mixing among multiple diabatic states, as analyzed further in Figure S5. The calculated electronic couplings are insensitive to the diabatization scheme, as the BoysOV diabatization gives very similar results (Figure S6) to the ER diabatization shown in Figure 6. The calculated electronic coupling between the LES and the LEPT state is roughly half of the energy splitting between the S_2_ and S_3_ states for both functionals at all *R*_PT_ values. This behavior is expected because all electronic coupling calculations were performed at the crossing point between the LES and the LEPT state.

**Figure 6:**
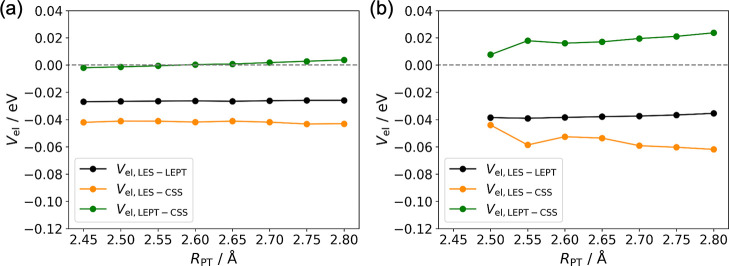
Electronic couplings among the LES, LEPT state, and CSS along the proton donor–acceptor distance *R*_PT_ calculated at the MECP geometry between the LES and the LEPT state using the (a) CAM-B3LYP and (b) ωB97XD functionals. ER diabatization was used for the electronic coupling calculations.

The main difference between the results obtained with the two functionals is that the electronic coupling between the LEPT state and the CSS changes sign as *R*_PT_ increases when using the CAM-B3LYP functional but retains the same sign for all *R*_PT_ values studied when using the ωB97XD functional. Note that the electronic wave functions obtained from electronic structure calculations, and thus the calculated electronic coupling, can have an arbitrary sign. We ensured that the relative signs of the wave functions are consistent for each *R*_PT_ value by monitoring the transition dipole moments of the three diabatic states, which should be continuous as *R*_PT_ changes. Therefore, the sign change of the electronic coupling between the LEPT state and the CSS with *R*_PT_ is not an arbitrary sign change from the electronic structure calculation, although its origin is unclear. Because the indirect vibronic coupling is proportional to the electronic coupling between the LEPT state and the CSS in the electronically nonadiabatic limit, this sign change may alter the constructive or destructive interference between the different coupling terms.

Using these electronic couplings, we calculated the direct, indirect, and total vibronic couplings between the |I0⟩ and |II0⟩ vibronic states in the electronically nonadiabatic limit, as shown in Figure 7. The direct coupling is qualitatively similar for both functionals and exhibits fast, monotonic decay with respect to *R*_PT_ due to the significant decrease of the overlap integral *S*_Iμ,IIν_ as *R*_PT_ increases. The indirect vibronic coupling exhibits nonmonotonic variation at shorter *R*_PT_ values and decays at larger *R*_PT_ for both functionals. As *R*_PT_ changes, the shape of the proton potential energy profiles changes significantly, which strongly affects the overlap integrals *S*_Iμ,*jξ*_ and *S*_*jξ*,IIν_ and the energy difference between the vibronic states, *E*_IIν_ – *E*_*jξ*_, leading to a more complex dependence of the indirect vibronic coupling on *R*_PT_.

**Figure 7:**
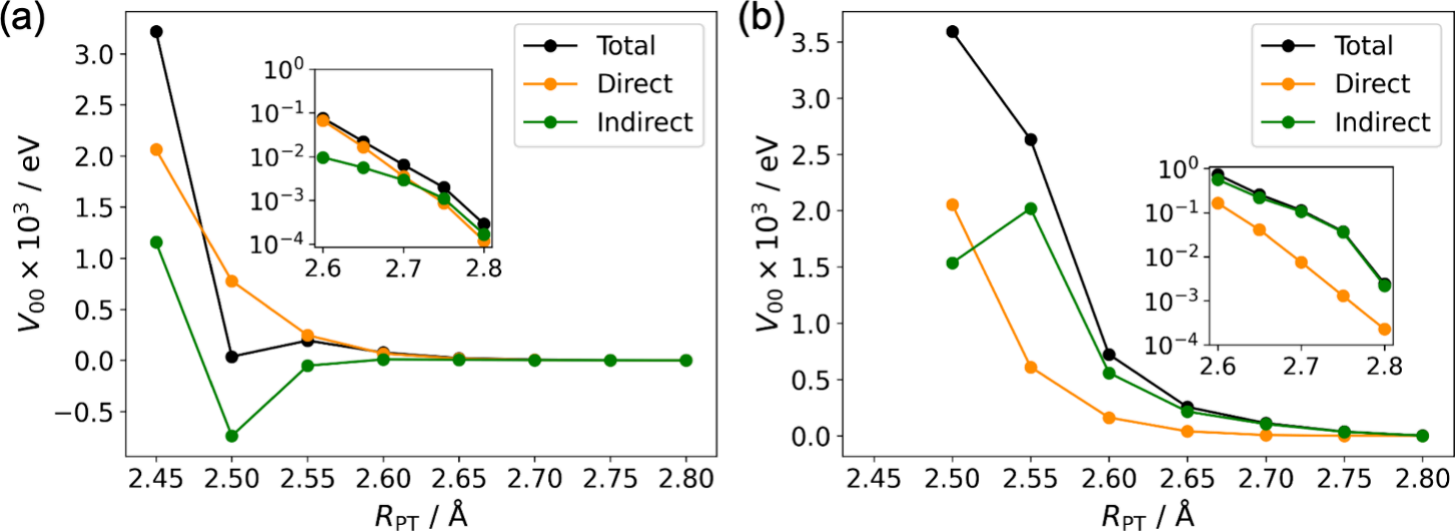
Direct, indirect, and total vibronic couplings between the |I0⟩ and |II0⟩ vibronic states along the proton donor–acceptor distance *R*_PT_ calculated using the (a) CAM-B3LYP and (b) ωB97XD functionals. For part (b), the point at 2.45 Å is not shown because the indirect contribution becomes numerically unstable. The insets show the same data plotted on a log scale for larger *R*_PT_. The numerical values are provided in Table S2.

The same qualitative conclusions hold for both functionals. First, at most *R*_PT_ values, the indirect coupling has comparable magnitude as or even larger magnitude than the direct coupling. This comparable magnitude arises because the electronic energies of the LES, LEPT state, and CSS are similar at the MECP geometries. Thus, the energy difference between the vibronic states, *E*_IIν_ – *E*_*jξ*_, can be very small for certain |*jξ*⟩ states, typically corresponding to ξ = 0 or 1 (Figures S1 and S2). As shown in eq 22, the indirect coupling is proportional to the inverse of this energy difference, and thus smaller *E*_IIν_ – *E*_*jξ*_ leads to larger indirect coupling. The indirect coupling also depends on the product of the overlap integrals *S*_Iμ,*jξ*_ and *S*_*jξ*,IIν_. For most cases, the |*j*1⟩ state, where ξ = 1, dominates the indirect vibronic coupling because the proton vibrational wave function for this state can delocalize over both wells in the double-well proton potential, leading to large proton vibrational wave function overlap with both the |I0⟩ and |II0⟩ vibronic states. The quantitative differences in the indirect couplings predicted by the CAM-B3LYP and ωB97XD functionals mainly arise from the differences in the CSS energy and the shapes of the proton potential energy profiles obtained from the two functionals.

For both functionals and at most *R*_PT_ values, the indirect coupling interferes constructively with the direct coupling, making the total vibronic coupling larger and facilitating the PCEnT reaction. The outliers are at *R*_PT_ = 2.50 and 2.55 Å for the CAM-B3LYP functional, where the indirect coupling interferes destructively with the direct coupling and hinders PCEnT. As *R*_PT_ changes, multiple quantities can change sign, altering the nature of the interference in terms of constructive versus destructive.

Figure 8 shows the comparison between the total vibronic coupling calculated using eq 22 or the dipole–dipole approximation in eq 23 for the triad. The absolute values are plotted in this figure on a log scale. For the CAM-B3LYP functional, the vibronic coupling calculated using the dipole–dipole approximation agrees well with the total vibronic coupling calculated using eq 22 for all *R*_PT_ values except at 2.50 Å. For the ωB97XD functional, the dipole–dipole approximation underestimates the total vibronic coupling at all *R*_PT_ values, resulting in an underestimation of the PCEnT rate constant, although the trend remains intact. We emphasize that the total vibronic coupling encompasses Coulomb, exchange, and indirect interactions, whereas the dipole–dipole approximation only includes the leading term of the Coulomb interaction. Our discussion above suggests that the indirect interaction makes a non-negligible contribution to the total vibronic coupling for both functionals. Therefore, the dipole–dipole approximation does not correctly describe the nature of the interaction between the LES and the LEPT state, and any agreement may be fortuitous for this system.

**Figure 8:**
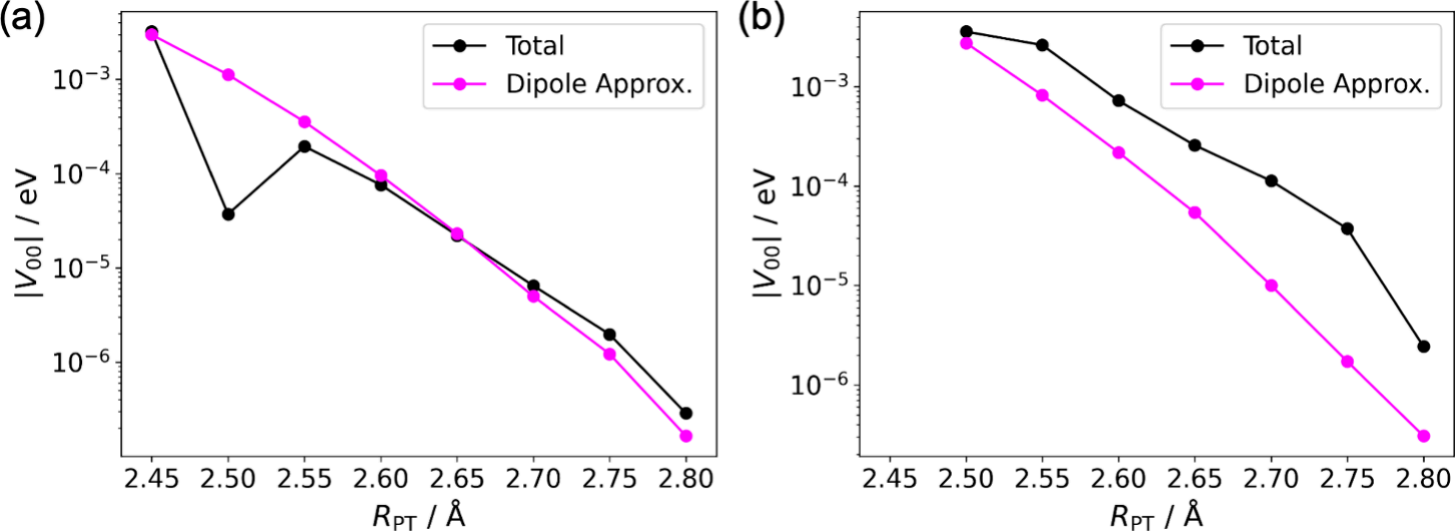
Absolute value of the total vibronic coupling between the |I0⟩ and |II0⟩ vibronic states along the proton donor–acceptor distance *R*_PT_ calculated using the general expression in eq 22 and the dipole–dipole approximation in eq 23 with the (a) CAM-B3LYP and (b) ωB97XD functionals.

### 4.3 When Should the Indirect Coupling Be Included?

Our analysis in the previous section reveals the limitation of the dipole–dipole approximation for PCEnT in the triad system, an intramolecular singlet–singlet process, and shows the importance of including indirect coupling terms in the vibronic coupling calculation. The main reason is that for the triad at the MECP geometry, the electronic energy of the CSS is very close to the electronic energies of the LES and LEPT state. As a result, some vibronic states associated with the CSS (mainly |*j*0⟩ and |*j*1⟩) become nearly degenerate with the reactant and product vibronic states corresponding to |I0⟩ and II0⟩. The proton potential energy profile for the CSS also has a lower barrier, making |*j*1⟩ delocalized enough to overlap significantly with both the reactant and product vibronic states. Other scenarios where indirect coupling must be considered include solution-phase intermolecular PCEnT, triplet–triplet PCEnT, and PCEnT in donor–bridge–acceptor systems.

Recently, the Hammarström group showed experimental evidence of intermolecular PCEnT occurring between a benzo­[ghi]­perylene donor and 3-hydroxyflavone acceptor at room temperature in solution.^12^ In this case, the energy donor and acceptor approach each other through diffusion, and PCEnT may occur at any energy donor–acceptor distance. To computationally estimate this intermolecular PCEnT rate constant, the PCEnT rate constant must be calculated at various distances and relative orientations between the energy donor and acceptor, followed by averaging over the radial and angular pair distribution function between them. The Coulomb, exchange, and indirect coupling terms have distinct distance and orientation dependence. For example, the dipole–dipole coupling decays as *R* ^–3^ with respect to the energy donor–acceptor distance *R*, whereas the exchange and indirect coupling terms decay exponentially with *R*. The dipole–dipole interaction depends on the relative orientation between the energy donor and acceptor through the orientation factor κ, whereas the orientation dependence in the exchange and indirect interactions mainly arises from electronic wave function overlap.^22^ Therefore, the relative contributions from these terms to the total vibronic coupling will strongly depend on the distance and orientation between the energy donor and acceptor. All terms must be explicitly considered to obtain the correct coupling over a wide range of distances and orientations.

For conventional triplet–triplet EnT, the Coulomb interaction in the direct coupling term vanishes because of the orthogonality between different spin states, and the total electronic coupling is dominated by exchange and indirect interactions. Both terms decay exponentially with the energy donor–acceptor distance. It has been shown that the magnitude of the second-order, indirect coupling is comparable with or even larger than the first-order term −*K* and thus must be included to accurately calculate the electronic coupling for Dexter-type EnT.^17,19^ We expect similar behavior for triplet–triplet PCEnT, where the magnitude of the indirect vibronic coupling $V_{\mu \nu }^{\left(2\right)}$ will be similar to the first-order term −*KS*_Iμ,IIν_, especially when a vibronic state |*jξ*⟩ is nearly degenerate with the states |Iμ⟩ and |IIν⟩ and also has substantial proton vibrational wave function overlap with them. Compared to conventional EnT, the near-degeneracy requirement is easier to be satisfied for PCEnT because the spacings between the electron–proton vibronic energy levels are generally much smaller than those between electronic states. However, the proton vibrational wave function associated with such an electronic state may not have significant vibrational overlap with both the |Iμ⟩ and |IIν⟩ vibronic states. The actual contribution of the indirect coupling will thus be system dependent. Although direct experimental evidence of triplet–triplet PCEnT is not yet available, Ramundo et al. hypothesized that it may be operating during the light-activated carbon monoxide release in porphyrin-flavonol hybrids.^49^

PCEnT in donor-bridge-acceptor systems is another example where the indirect coupling may be important. In a donor-bridge-acceptor system, there are more possible virtual intermediate electronic states |*j*⟩, such as the local excited states on the bridge, the charge transfer states between the donor and the bridge, and the charge transfer states between the acceptor and the bridge. All electron–proton vibronic states associated with these electronic states can act as possible virtual intermediate vibronic states. More possible virtual intermediate states result in more possible indirect interaction pathways and thus greater potential for quantum interference between these pathways. In this case, the third- and even fourth-order terms in perturbation theory could also be important, as shown for conventional EnT.^18,19^

## 5 Conclusion

Herein, we derived an analytical expression for the vibronic coupling in PCEnT processes that encompasses both direct and indirect coupling between the reactant and product vibronic states. The direct coupling includes both Coulomb and exchange terms, although for singlet–singlet energy transfer at relatively large energy donor–acceptor distances, the dipole–dipole Coulomb term is expected to be dominant. For the indirect coupling, the interaction between the reactant and product vibronic states is mediated by at least one virtual intermediate vibronic state, which likely has charge transfer character. We provide a general algorithm and procedure to calculate the input quantities for this general vibronic coupling expression.

As an example, we calculated the vibronic coupling between the LES and LEPT state in the anthracene−phenol−pyridine triad system as a function of the proton donor–acceptor distance. We found that only the CSS needs to be considered as a possible virtual intermediate state. The indirect coupling has comparable or even larger magnitude than the direct coupling at most proton donor–acceptor distances, and the indirect coupling can interfere either constructively or destructively with the direct coupling. This analysis suggests that both Förster and Dexter mechanisms are contributing to this PCEnT process.

The theory and methods developed in this paper are applicable to other PCEnT systems. The general expression for the vibronic coupling will be especially important for solution phase intermolecular PCEnT, triplet–triplet PCEnT, and PCEnT in donor−bridge−acceptor systems, where the indirect coupling may become more important. This development builds on and complements the recently developed nonadiabatic PCEnT theory and provides a useful tool for computing a key parameter in the PCEnT rate constant. Thus, this work adds to the foundation for future studies of a wide range of PCEnT systems.

## Supplementary Material



## Data Availability

The data that support the findings of this study are openly available on Zenodo at https://doi.org/10.5281/zenodo.19826935. The MECP optimization code is available on Github at https://github.com/kcui1995/aseMECP.

## Notes

The calculated proton potentials and vibrational wave functions associated with the LES, LEPT state, and CSS at the MECP geometry at different proton donor–acceptor distances; vibronic coupling results using the BoysOV diabatization method (PDF)
