# Supplementary material for: General Expression for Vibronic Coupling in Proton-Coupled Energy Transfer
Source: J Chem Theory Comput. 2026 May 24;22(11):5674–85. doi: 10.1021/acs.jctc.6c00443 (PMC13255174; doi:10.1021/acs.jctc.6c00443)
Supplement: Supplementary file 1 [file ct6c00443_si_001.pdf]

Supporting Information
General Expression for Vibronic Coupling in Proton-Coupled Energy Transfer

Kai Cui and Sharon Hammes-Schiffer*

Department of Chemistry, Princeton University, Princeton, NJ 08544, United States
*Email: shs566@princeton.edu

S1 Supporting Figures and Tables

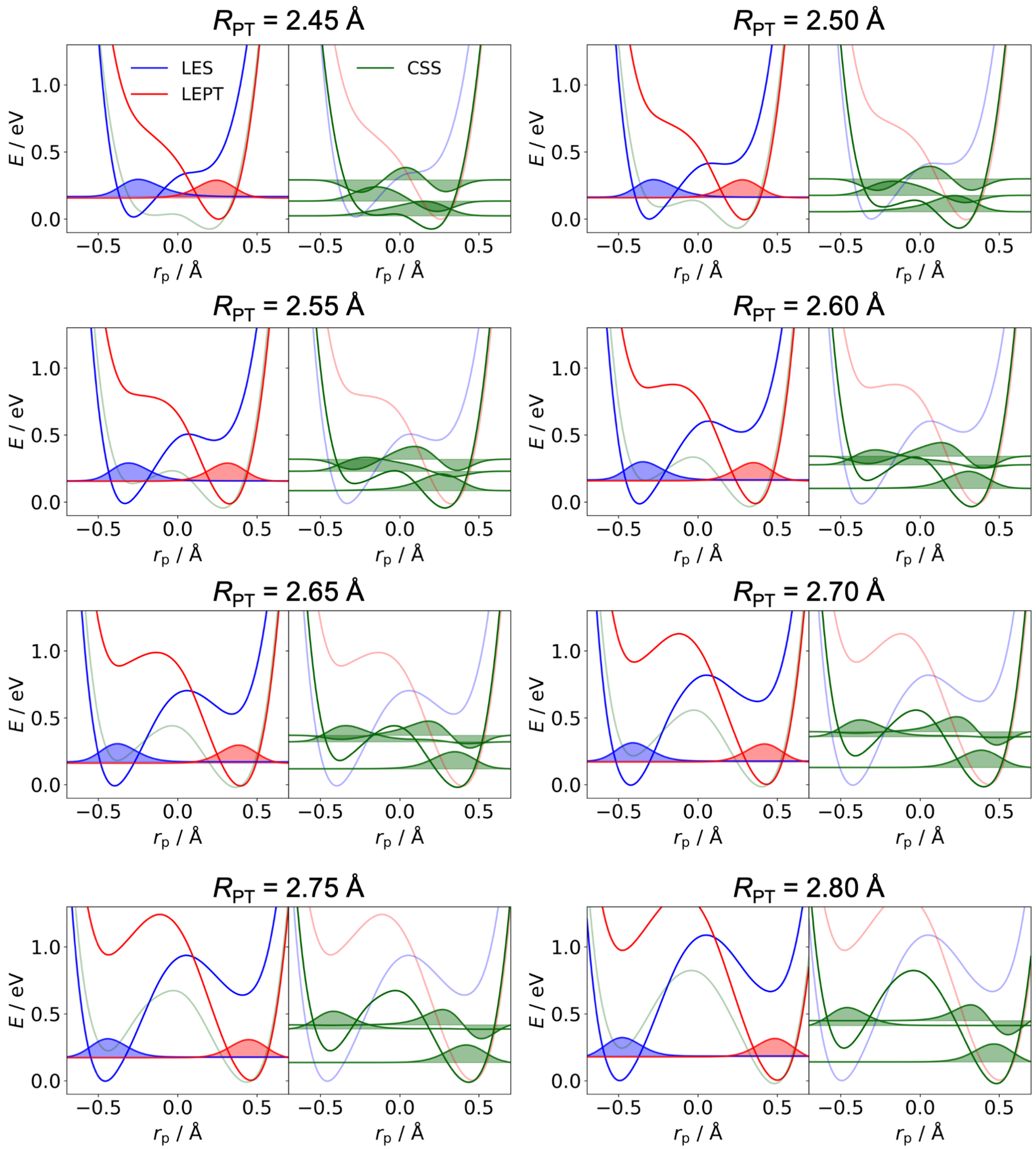

Figure S1: Proton potential energy profiles and proton vibrational wave functions associated with the LES, LEPT state, and CSS at the MECP geometry between the LES and the LEPT state at different proton donor–acceptor distances computed using the CAM-B3LYP functional.

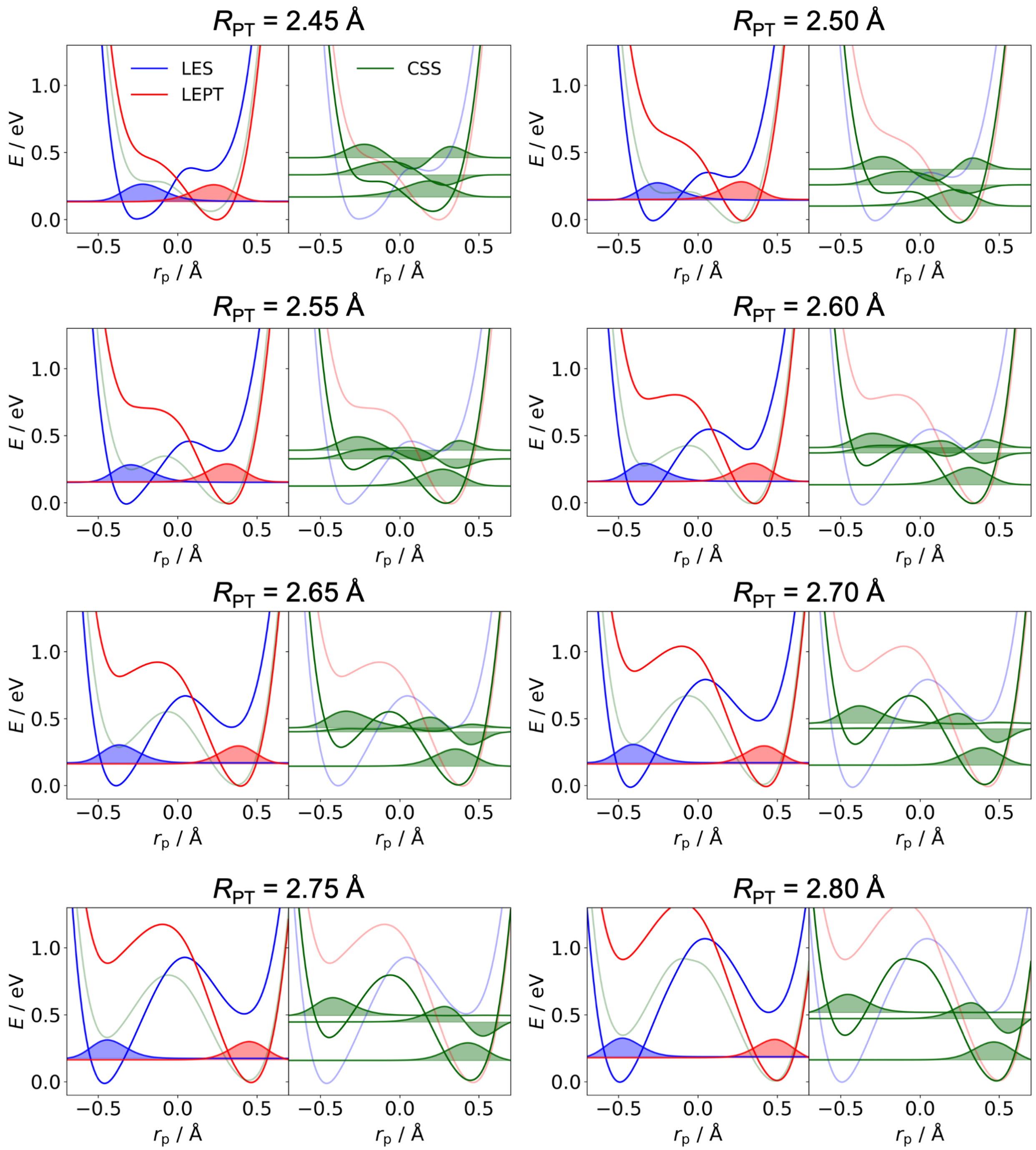

Figure S2: Proton potential energy profiles and proton vibrational wave functions associated with the LES, LEPT state, and CSS at the MECP geometry between the LES and the LEPT state at different proton donor–acceptor distances computed using the ωB97XD functional.

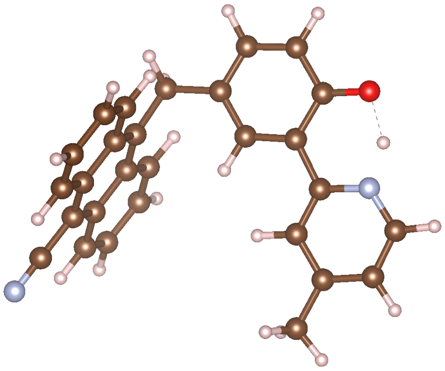

Figure S3: A representative MECP geometry between the LES and the LEPT state optimized using the CAM-B3LYP functional with the proton donor–acceptor distance R_{PT} constrained at 2.60 Å. The transferring proton is placed at the intersection point between the proton potential energy profiles for the LES and LEPT state at this geometry.

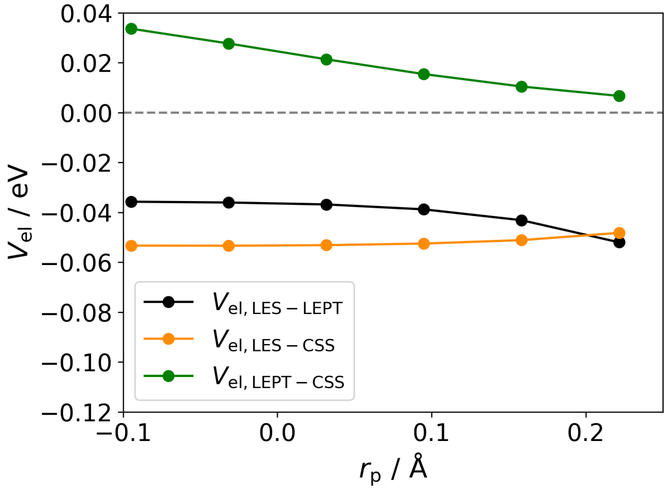

Figure S4: Electronic couplings among the LES, LEPT state, and CSS along the one-dimensional proton coordinate r_{p}. These couplings were calculated at the MECP geometry between the LES and the LEPT state at $R_{\mathrm{PT}} = 2.60$ Å using the ωB97XD functional. ER diabatization was used for the electronic coupling calculations. Note that when $r_{\mathrm{p}} < -0.1$ Å and $r_{\mathrm{p}} > 0.25$ Å, other excited states (e.g., LES2 and LEPT2) become close in energy to and mix with the LES, LEPT state, and CSS, and the diabatization methods become less reliable. The averaged electronic couplings over this r_{p} range agree well with the electronic couplings calculated at the crossing point of the two one-dimensional proton potential energy profiles.

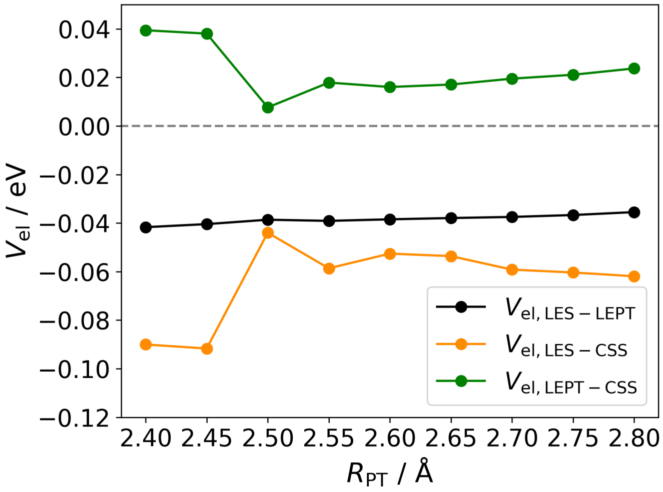

Figure S5: Electronic couplings among the LES, LEPT state, and CSS along the proton donor–acceptor distance R_{PT} calculated at the MECP geometry between the LES and the LEPT state using the ωB97XD functional. The electronic coupling calculations were performed with ER diabatization at the crossing point between the proton potential energy curves. The data at $R_{\mathrm{PT}} \geq 2.50$ Å are the same as Figure 6b in the main text, whereas the data at $R_{\mathrm{PT}} < 2.50$ Å are not shown in the main text because they are less reliable. For $R_{\mathrm{PT}} \geq 2.50$ Å, the S_1 state with CSS character is well-separated from the S_2 and S_3 states, which are superpositions of the LES and LEPT state, whereas for $R_{\mathrm{PT}} \leq 2.45$ Å, all three diabatic states mix at the crossing point between the proton potential energy curves. This mixing could lead to inconsistencies in the diabatization procedure.

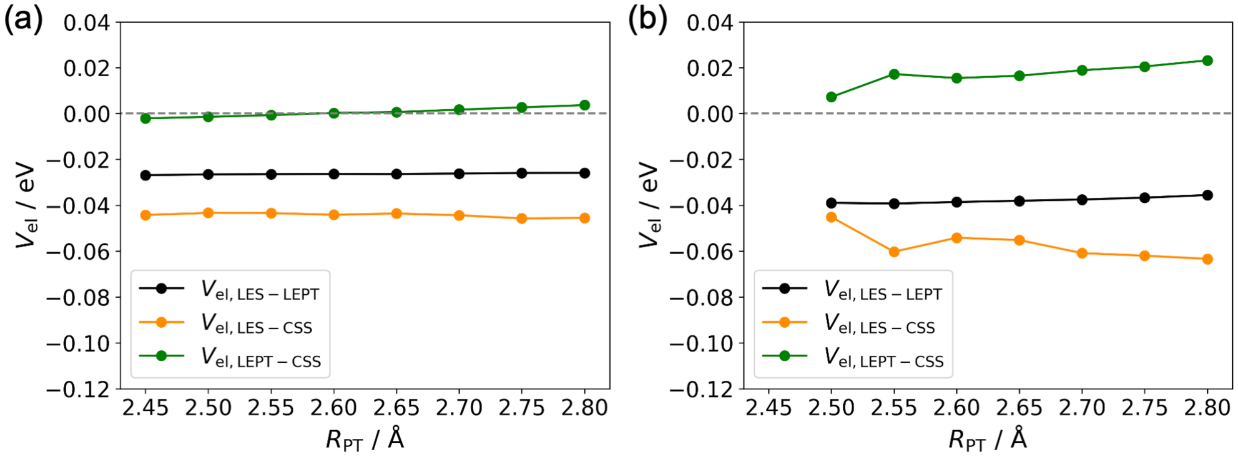

Figure S6: Electronic couplings among the LES, LEPT state, and CSS along the proton donor-acceptor distance R_{PT} calculated at the MECP geometry between the LES and the LEPT state using the (a) CAM-B3LYP and (b) ωB97XD functionals. BoysOV dibatization was used for the electronic coupling calculations, in contrast to the ER diabatization used to produce Figure 6 in the main paper.

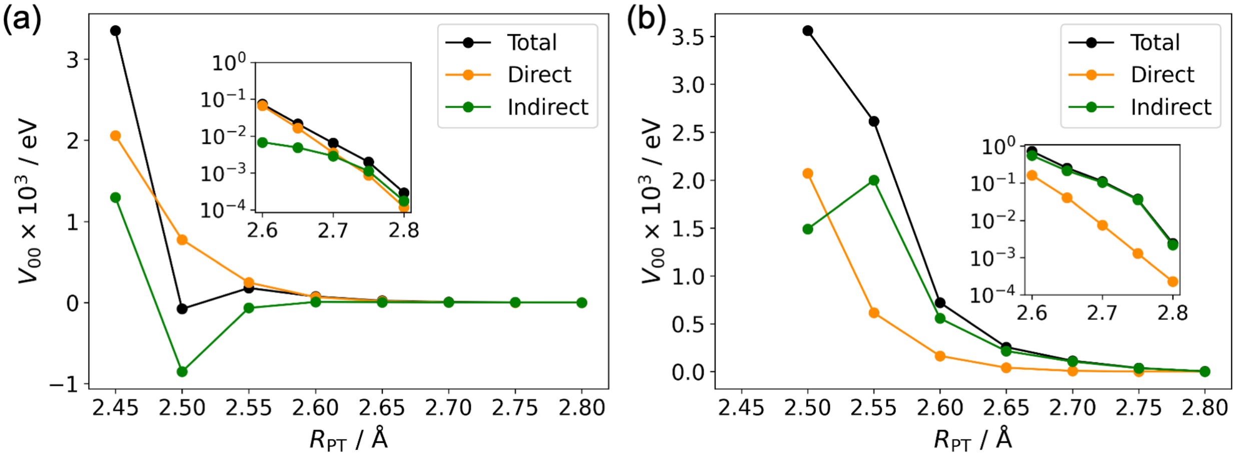

Figure S7: Direct, indirect, and total vibronic couplings between the $|I0\rangle$ and $|II0\rangle$ vibronic states along the proton donor-acceptor distance R_{PT} calculated using the (a) CAM-B3LYP and (b) ωB97XD functionals. The insets show the same data plotted on a log scale for larger R_{PT}. The numerical values are provided in Table S3. BoysOV diabatization was used for the electronic coupling calculation, in contrast to the ER diabatization used to produce Figure 7 in the main paper.

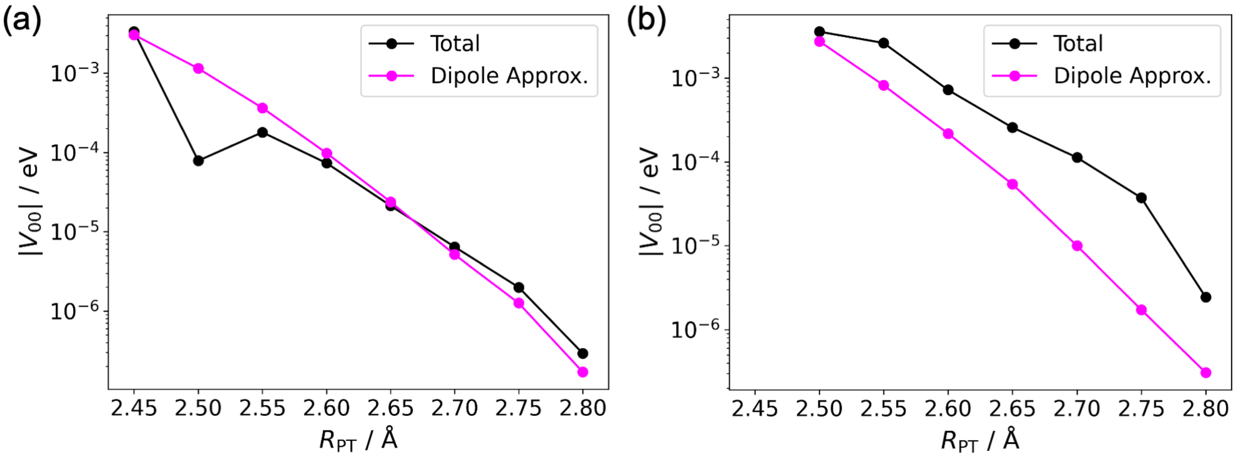

Figure S8: Absolute value of the total vibronic coupling between the $|I0\rangle$ and $|II0\rangle$ vibronic states along the proton donor-acceptor distance R_{PT} calculated using the general expression in Eq. (22) and the dipole-dipole approximation in Eq. (23) with the (a) CAM-B3LYP and (b) ωB97XD functionals. BoysOV dibatization was used for the electronic coupling calculation, in contrast to the ER diabatization used to produce Figure 8 in the main paper.

Table S1: Direct, Indirect, and Total Vibronic Couplings between the $|I0\rangle$ and $|II0\rangle$ Vibronic States of the Triad Calculated Using the CAM-B3LYP Functional [a]

Vibronic Coupling / eV	
Direct	-2.52×10^{-5}
Indirect via CSS	-6.19×10^{-5}
Indirect via LES2	4.39×10^{-7}
Indirect via LEPT2	6.42×10^{-7}
Indirect via RCSS	1.84×10^{-7}
Total	-8.59×10^{-5}

[a] All electronic couplings between the electronic states are assumed to be the same, with a value of 0.01 eV, for this analysis.

Table S2: Direct, Indirect, and Total Vibronic Couplings between the $|\mathrm{I0}\rangle$ and $|\mathrm{II0}\rangle$ Vibronic States of the Triad Calculated Using ER Diabatization and the CAM-B3LYP and ωB97XD Functionals

CAM-B3LYP				
R_{PT} / Å	Direct	Indirect	Total	Interference
2.45	2.06×10^{-3}	1.16×10^{-3}	3.22×10^{-3}	constructive
2.50	7.76×10^{-4}	-7.39×10^{-4}	3.71×10^{-5}	destructive
2.55	2.46×10^{-4}	-5.23×10^{-5}	1.94×10^{-4}	destructive
2.60	6.64×10^{-5}	9.58×10^{-6}	7.60×10^{-5}	constructive
2.65	1.65×10^{-5}	5.59×10^{-6}	2.21×10^{-5}	constructive
2.70	3.55×10^{-6}	2.92×10^{-6}	6.47×10^{-6}	constructive
2.75	8.70×10^{-7}	1.11×10^{-6}	1.98×10^{-6}	constructive
2.80	1.19×10^{-7}	1.68×10^{-7}	2.87×10^{-7}	constructive
ωB97XD				
R_{PT} / Å	Direct	Indirect	Total	Interference
2.50	2.05×10^{-3}	1.54×10^{-3}	3.59×10^{-3}	constructive
2.55	6.12×10^{-4}	2.02×10^{-3}	2.63×10^{-3}	constructive
2.60	1.63×10^{-4}	5.61×10^{-4}	7.24×10^{-4}	constructive
2.65	4.06×10^{-5}	2.17×10^{-4}	2.57×10^{-4}	constructive
2.70	7.53×10^{-6}	1.05×10^{-4}	1.13×10^{-4}	constructive
2.75	1.30×10^{-6}	3.59×10^{-5}	3.72×10^{-5}	constructive
2.80	2.28×10^{-7}	2.21×10^{-6}	2.44×10^{-6}	constructive

Table S3: Direct, Indirect, and Total Vibronic Couplings between the $|\mathrm{I}0\rangle$ and $|\mathrm{II}0\rangle$ Vibronic States of the Triad Calculated Using BoysOV Diabatization and the CAM-B3LYP and ωB97XD Functionals

CAM-B3LYP				
R_{PT} / Å	Direct	Indirect	Total	Interference
2.45	2.06×10^{-3}	1.30×10^{-3}	3.35×10^{-3}	constructive
2.50	7.74×10^{-4}	-8.52×10^{-4}	-7.86×10^{-5}	destructive
2.55	2.46×10^{-4}	-6.69×10^{-5}	1.79×10^{-4}	destructive
2.60	6.66×10^{-5}	6.80×10^{-6}	7.34×10^{-5}	constructive
2.65	1.64×10^{-5}	4.89×10^{-6}	2.13×10^{-5}	constructive
2.70	3.55×10^{-6}	2.89×10^{-6}	6.44×10^{-6}	constructive
2.75	8.70×10^{-7}	1.13×10^{-6}	2.00×10^{-6}	constructive
2.80	1.19×10^{-7}	1.74×10^{-7}	2.92×10^{-7}	constructive
ωB97XD				
R_{PT} / Å	Direct	Indirect	Total	Interference
2.50	2.07×10^{-3}	1.49×10^{-3}	3.56×10^{-3}	constructive
2.55	6.15×10^{-4}	2.00×10^{-3}	2.61×10^{-3}	constructive
2.60	1.64×10^{-4}	5.57×10^{-4}	7.21×10^{-4}	constructive
2.65	4.08×10^{-5}	2.16×10^{-4}	2.56×10^{-4}	constructive
2.70	7.54×10^{-6}	1.05×10^{-4}	1.13×10^{-4}	constructive
2.75	1.30×10^{-6}	3.60×10^{-5}	3.73×10^{-5}	constructive
2.80	2.29×10^{-7}	2.22×10^{-6}	2.45×10^{-6}	constructive
